# Microbial Diversity and Biodegradation Mechanism of Microorganisms in the Dingtao M2 Tomb

**DOI:** 10.3390/ijms252212270

**Published:** 2024-11-15

**Authors:** Yu Wang, Cen Wang, Lilong Hou, Xinyu Yang, Chenghao Li, Shengkuan Cui, Cuilian Ma, Ling Wang, Lu Zhang, Yuanyuan Liu, Hong Guo, Jiao Pan

**Affiliations:** 1 Key Laboratory of Archaeomaterials and Conservation, Ministry of Education, University of Science and Technology Beijing, Beijing 100083, China; d202310766@xs.ustb.edu.cn (Y.W.); 2120211255@mail.nankai.edu.cn (C.W.); 2 Institute for Cultural Heritage and History of Science & Technology, University of Science and Technology Beijing, Beijing 100083, China; 3 College of Life Sciences, Nankai University, Tianjin 300071, China; 2120221471@mail.nankai.edu.cn (L.H.); alicexyyang@163.com (X.Y.); 4 Shandong Provincial Institute of Cultural Relics and Archaeology, Jinan 250001, China; lichenghaohy@163.com (C.L.); SDKGCSK@163.com (S.C.); 5 Preservation Research Center of the Mausoleum of the Dingtao King, Heze 274100, China; mcl14707@163.com (C.M.); 17863206119@163.com (L.W.); 13305307331@163.com (L.Z.); dycliangshan@163.com (Y.L.)

**Keywords:** the Dingtao M2 tomb, microbial diversity analysis, biodegradation mechanism, *Dacrymyces stillatus*, *Talaromyces pinophilus*

## Abstract

The Dingtao M2 tomb, the largest and best-preserved imperial “Huangchangticou” tomb in China, holds great significance for its conservation. Currently, varying degrees of microbial degradation are occurring on the surfaces of the M2 tomb. This study aimed to determine the microbial diversity of the M2 tomb and its surrounding environment during July 2021 and August 2022. High-throughput metagenomic sequencing revealed that the dominant fungus on the surface of the tomb chamber was *Dacrymyces stillatus* (DTT1) in July 2021, which changed to *Talaromyces pinophilus* (DTT2) in August 2022. Enzymatic activities for cellulose and lignin degradation suggested that DTT1 has high levels of manganese peroxidase, lignin peroxidase, laccase, and cellulase. The wood of the tomb contained higher levels of Fe^2+^ and Ca^2+^, and experiments with different concentration gradients of these ions in the culture medium revealed that DTT1 exhibited greater activity of cellulose and lignin degradation in environments with higher concentrations of Fe^2+^ and Ca^2+^. DTT2 degraded both cellulose and lignin. Lastly, a laboratory plate inhibition experiment demonstrated that isothiazolinone fungicide had a significant fungicidal effect on these two dominant fungi. This study provides valuable data and a theoretical basis for the preservation of the M2 tomb and other wooden cultural relics.

## 1. Introduction

Cultural relics have been left over by humans during historical development and have been considered the precious historical and cultural heritage of humanity [1]. Some cultural relics have been buried for many years and have been exposed to complex environmental conditions, leading to varying degrees of damage [2]. Among these, the corrosion and damage caused by microorganisms to cultural relics are receiving increasing attention from people [3,4]. The destruction of cultural relics by microorganisms is a complex process primarily driven by microbe related biological activities [5,6]. However, limited research has been conducted to investigate the mechanisms underlying the microbial destruction of cultural relics. While, previously, the chemical reactions were considered a cause of the destruction, it is now widely accepted that bacteria, algae, and fungi can cause significant damage to cultural heritage sites through their secretion of enzymes and organic substances produced during metabolism [7]. This finding is especially true for organic cultural relics, which provide nutrients for microbial growth. Fungi, in particular, play a significant role in damaging cultural relics due to their complex metabolic activities, ability to degrade various organic materials, and production of organic acids and pigments that corrode cultural relics. Therefore, the damage caused by fungi to cultural relics warrants special attention [8,9].

Wooden cultural relics are common and important organic cultural artifacts, often found in the form of ships, tombs, houses, etc. [10]. They served as valuable physical materials and carriers of ancient human civilization. Wooden cultural relics can be categorized as either dry or water-saturated [11,12]. Dry wooden relics have reached a balance with their environment when unearthed or watered, are less affected by water content, and maintain a good state. Water-saturated wooden artifacts, on the other hand, are filled with water during underground or underwater burial processes and have increased moisture content, such as sunken ships and warships salvaged from various locations like China, Sweden, and Italy [13,14,15]. The chemical composition and moisture content of wood are commonly used to assess the preservation status of waterlogged wooden cultural relics. The holocellulose in the cell walls of waterlogged wooden artifacts degrades, causing its proportion to decrease while the proportion of lignin increases. Simultaneously, as the cell wall degrades, a large number of holes are generated, filling the original area of the cell wall with water [16]. Typically, the moisture content of fresh wood is approximately 60–120%, but, in waterlogged wooden artifacts, it can exceed 700%. The higher the moisture content, the more severe the degradation of waterlogged wooden artifacts, and the worse their preservation condition becomes [11,12]. Due to these factors, it is challenging for waterlogged wooden artifacts to maintain a good state after excavation. Without proper preservation, they may experience severe shrinkage, cracking, and deformation, ultimately leading to irreparable damage [17].

Fungi are the most effective and abundant wood decomposers. Their hyphae provide them with the advantage of spreading their growth over most prokaryotes. They are generally classified according to the type of biopolymer they prefer to degrade. White-rot fungi break down cellulose, hemicellulose, and lignin, whereas brown-rot fungi break down cellulose and hemicellulose, leaving modified lignin. Another type of rot disease, mainly caused by ascomycetes, is soft rot disease. This disease causes the degradation of cellulose and hemicellulose, forming conical and spongy pores [18,19,20].

The tomb of the Dingtao King is located in Dingtao District, Heze City, Shandong Province. Experts believe it dates back to the late Western Han Dynasty, making it over 2000 years. The M2 tomb is part of the extensive “Huangchangticoutomb”, and the associated “Huangchangticou” burial system was reserved for high-ranking nobles of the Han Dynasty. M2 is the largest of three sealed tombs at the site and is currently the most significant single Han Dynasty “Huangchangticou” burial system excavated in China [21,22]. Its grand scale, precise structure, and unique design make it the largest and best-preserved imperial “Huangchangticou” tomb in China. The discovery of the Dingtao Han Tomb was a significant event for the Chinese archeological community. It not only showcased the glorious civilization of the ancient city of Dingtao to the world, but it also confirmed the prevalence of honorable burials among the Han people. There were significant changes in the types, shapes, and styles of the tombs compared to the previous generation. This discovery holds positive and significant historical value for studying the social structure of the Han Dynasty, people’s tomb consciousness, cultural concepts, and attitudes toward life and death.

The preservation of large wooden cultural relics is a global challenge. A significant amount of wood was unearthed from the M2 tomb, and, after being waterlogged for over 2000 years, many issues such as wood cracking and salt damage arose during excavation. Currently, there are varying degrees of microbial diseases present on the surface of the M2 tomb. The primary objective of this study was to utilize high-throughput sequencing to identify the predominant microorganisms present on the surface of the M2 tomb. Subsequently, enzyme activity assays were conducted to investigate the degradation capabilities of these microorganisms, as well as the mechanisms behind their growth on the tomb’s surface. Lastly, fungicidal experiments were conducted to determine the most effective fungicides. This study offers fundamental data and recommendations for the future preservation of the M2 tomb, serving as a valuable reference for addressing microbial diseases in other wooden cultural relics. It holds significance and value in the realm of cultural relic preservation.

## 2. Results

### 2.1. Scanning Electron Microscope (SEM) Results of Surface Samples of the M2 Tomb

**Figure 1:**
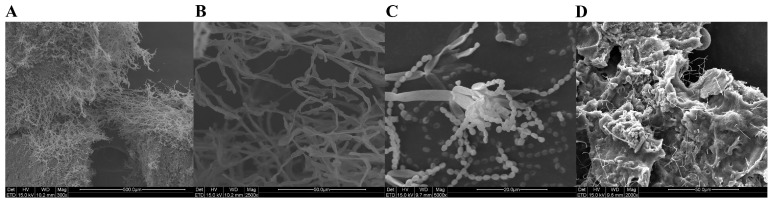
SEM results of the surface of the Dingtao M2 tomb: (**A**) DTTS1; (**B**) DTTS2; (**C**) DTM2.1; and (**D**) DTM2.4.

The SEM results indicate that the white plaque on the surface of the M2 tomb in July 2021 was formed by the interweaving of hyphae (Figure 1A), with a clear hyphal structure and no spores (Figure 1B). In the yellow-green plaque samples taken in August 2022, a large number of hyphae and spores were observed under SEM. The structure of the sporangium can also be seen (Figure 1C), which differs from the hyphal structure observed in the samples of July 2021, indicating a change in the main disease-causing microorganisms on the surface of the tomb. Only a small number of microbial structures were observed in the DTM2.4 sample (Figure 1D).

### 2.2. Microbial Composition on the Surface and Surrounding Environment of the M2 Tomb

**Figure 2:**
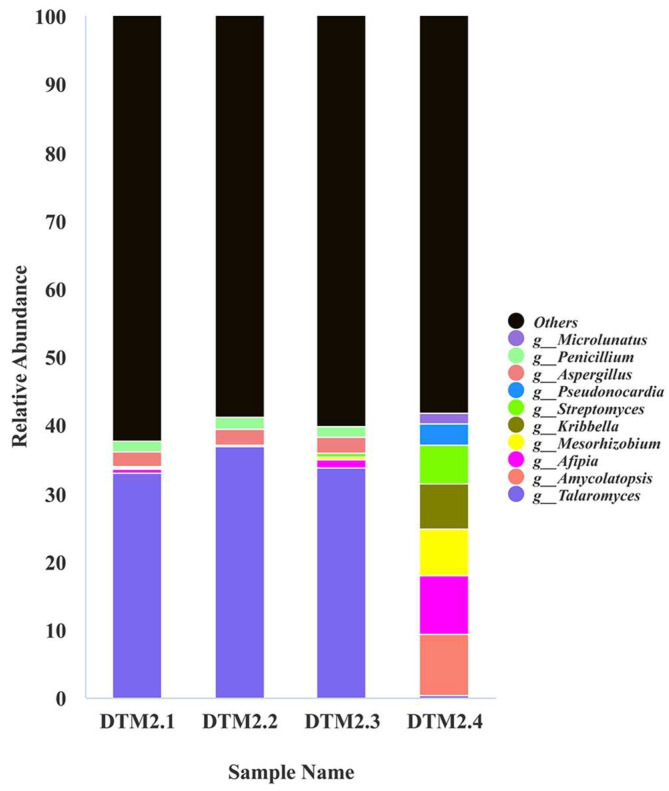
Relative abundance of microorganisms at the genus level on the surface of the Dingtao M2 tomb.

High-throughput sequencing results revealed that the eukaryotes present on the tomb surface in July 2021 were primarily from Basidiomycota (Figure S1A) and *Dacrymyces*, with an average relative abundance of 99.8%. Metagenomic sequencing analyses of samples (DTM2.1–DTM2.4) collected in August 2022 showed that the highest relative abundance of Ascomycota at the phylum level was observed in DTM2.1, DTM2.2, DTM2.3, with contents of 67.37%, 75.58%, and 69.19%, respectively. In contrast, the relative abundance of Ascomycota in the DTM2.4 sample was only 0.69%. The predominant phyla in the DTM2.4 sample were Actinobacteriota, accounting for 61.78%, and Proteobacteria, accounting for 24.39%. Additionally, all four samples contained Acidobacteria and Basidiomycota (Figure S1B). At the genus level, the highest content in the DTM2.1–DTM2.3 samples belonged to the genus *Talaromyces*, with relative contents of 32.98%, 36.99%, and 33.77%, respectively. The DTM2.4 sample had lower levels of *Talaromyces* (only 0.32%) but higher levels of *Amycolatopsis*, *Afipia*, *Mesorhizobium*, *Kribbella*, and *Streptomyces*, with relative contents of 8.97%, 8.75%, 6.73%, 6.58%, and 5.73%, respectively (Figure 2).

A soil microbial sample was collected from the surface of earthen ruins for 18S high-throughput sequencing analysis. The sequencing results showed that the fungi on the surface of the earthen ruins belonged to phylum Ascomycota, with some Basidiomycota. At the genus level, the predominant fungi belonged to Hypocreales (Figure S2B,C), and the microorganisms found on the earthen sites were distinct from those present on the wood.

### 2.3. Database Annotation

**Figure 3:**
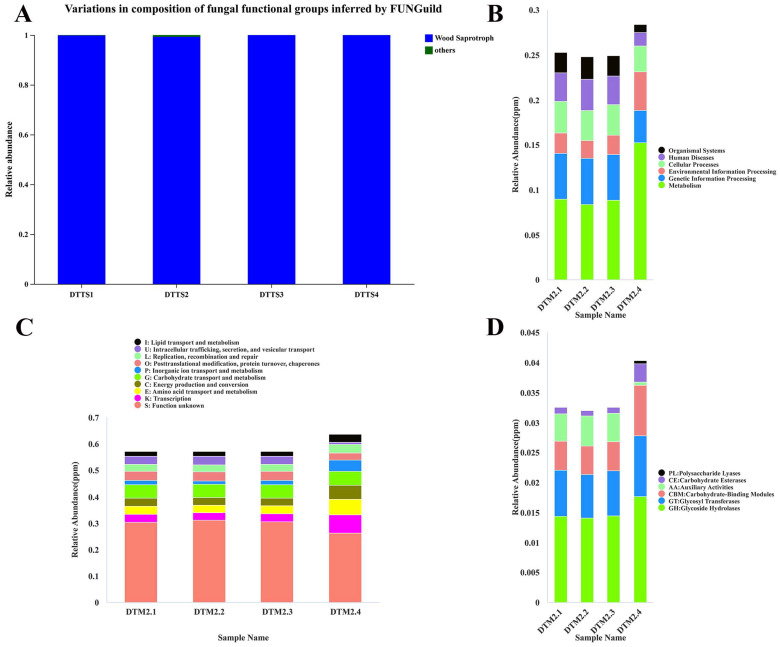
Database annotation results of each functional annotation: (**A**) FUNGuild function classification statistic histogram in high-throughput sequencing; (**B**) relative abundance of KEGG database at level 1 in metagenome sequencing; (**C**) relative abundance of eggNOG database at level1 in metagenome sequencing; and (**D**) relative abundance of CAZy database at level1 in metagenome sequencing.

We annotated the sequencing results in different databases to explore the potential degradation mechanisms of fungi in the M2 tomb. The FUNGuild annotation results indicated that *Dacrymyces* are wood saprotrophs (Figure 3A). According to the KEGG database level 1 annotation analysis, gene abundance in the metabolic pathway was the highest (Figure 3B). Annotations of the functions related to wood degradation at various levels were analyzed and compared to the KEGG Orthology (KO) database to identify relevant functions, such as beta-glucosidase (K05349) and alpha-glucosidase (K01187), with average relative abundances of 0.068% and 0.048%, respectively (Table S1). The annotation results at Level 1 of the eggNOG database revealed that transport, amino acid transport and metabolism, energy generation and conversion, and carbohydrate transport and metabolism were relatively high in abundance (Figure 3C). At the Orthologous Groups (OG) level, functions related to wood degradation, such as cellulase and laccase, were compared (Table S2). The Carbohydrate-Active enZYmes Database (CAZy) annotation results showed that levels of glycoside hydrolases (GHs), glycosyltransferases (GTs), carbohydrate-binding modules (CBMs), auxiliary oxidoreductase (AAs), carbohydrate esterases (CEs), and polysaccharide lyases (PLs) differed from high to low in the four samples of DTM2.1–DTM2.4 (Figure 3D).

### 2.4. Isolation, Purification, and Colony Morphology of Predominant Fungi

**Figure 4:**
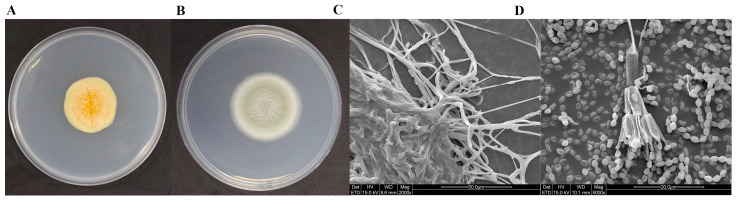
The growth morphology of dominant fungi on the surface of the M2 tomb: (**A**) DTT1 cultured at 25 °C light condition for 15 days on the PDA medium; (**B**) DTT2 cultured at 28 °C light condition for 5 days on the PDA medium; (**C**) SEM observation of DTT1; and (**D**) SEM observation of DTT2.

Based on the high-throughput and metagenomic sequencing results, it can be concluded that the predominant fungi on the surface of the M2 tomb are *Dacrymyces* and *Talaromyces*. We acquired *Dacrymyces stillatus* (ATCC 56530) from the American Type Culture Collection and designated it as DTT1. Observations of its growth morphology on a PDA medium indicated yellow-colored colonies that were closely integrated with the culture medium (Figure 4A). An SEM analysis revealed that DTT1 did not produce spores on the PDA medium, with clustered hyphae (Figure 4C). A strain of *Talaromyces* was isolated, purified, and named DTT2, identified as *T. pinophilus* through molecular methods. The growth morphology of a single-colony culture showed it to be green on a PDA medium with white edges and sporulation (Figure 4B). An SEM analysis revealed the production of spherical spores by DTT2, with the conidial stem as a symmetric bicyclic broom branch (Figure 4D).

### 2.5. Determination of Cellulose Lignin Degradation Capability of Predominant Fungi

**Figure 5:**
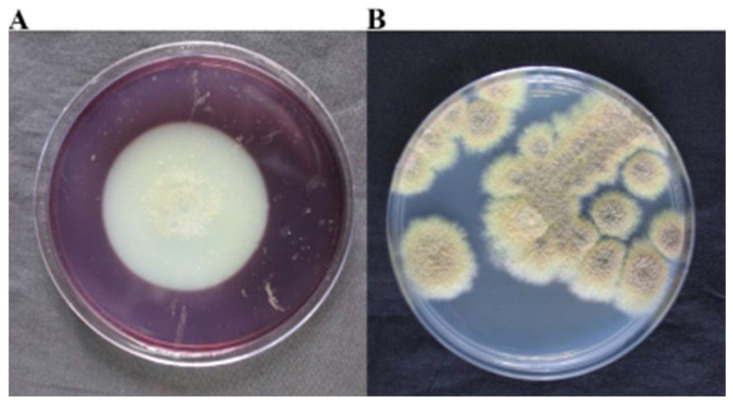
Determination of cellulose and lignin degradation capacity of DTT2: (**A**) The growth on CMC medium, incubated at 28 °C for 5 days; (**B**) the growth on PDA guaiacol medium, incubated at 28 °C for 5 days.

The enzyme activities of manganese peroxidase (MnP), lignin peroxidase (LiP), laccase (Lac), and cellulase of DTT1 were tested at various cultivation times. The data showed a strong cellulose lignin degradation capability of DTT1 (Table 1). DTT2 culture, growing on the CMC medium, showed the degradation of cellulose, leading to the formation of transparent circles when stained with the potassium iodide solution (Figure 5A). When grown on PDA, guaiacol turned brown, suggesting its potential to degrade lignin (Figure 5B).

### 2.6. Effect of Various Conditions on Enzyme Activity of Dacrymyces

**Figure 6:**
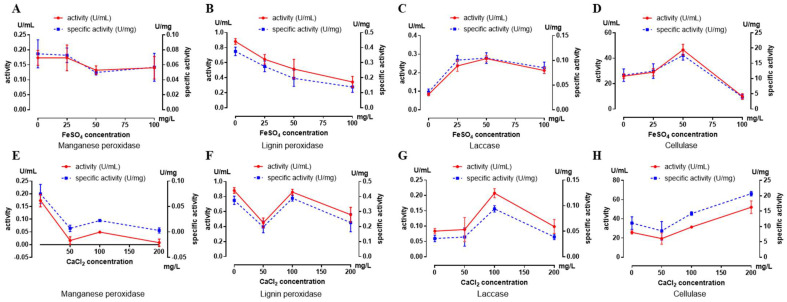
Effect of Fe^2+^ and Ca^2+^ on the enzyme and specific enzyme activities of DTT1: (**A**) Fe^2+^ vs. manganese peroxidase; (**B**) Fe^2+^ vs. lignin peroxidase; (**C**) Fe^2+^ vs. laccase; (**D**) Fe^2+^ vs. cellulase; (**E**) Ca^2+^ vs. manganese peroxidase; (**F**) Ca^2+^ vs. lignin peroxidase; (**G**) Ca^2+^ vs. laccase; and (**H**) Ca^2+^ vs. cellulase.

X-ray fluorescence and diffraction were used to analyze the physical and chemical properties of the tomb wood. The tomb chamber wood contained significant amounts of Fe and Ca, with average contents of 65.5% and 2.2%, respectively (Table S3). The X-ray diffraction results indicated the presence of compounds such as Fe_3_(PO_4_)_2_·3H_2_O and C_2_CaO_4_·2H_2_O (Figure S3). As the tomb surfaces may contain higher levels of Fe^2+^and Ca^2+^, the effects of these metal ions on the enzymatic activity and specific enzymatic activity of manganese peroxidase, lignin peroxidase, laccase, and cellulase in *Dacrymyces* was investigated. The data revealed that the enzyme activities of manganese peroxidase and lignin peroxidase decreased at high concentrations of Fe^2+^ and Ca^2+^, while the enzyme activities of laccase and cellulase increased to some extent (Figure 6).

### 2.7. Sensitivity of Dominant Fungi to Different Fungicides

**Figure 7:**
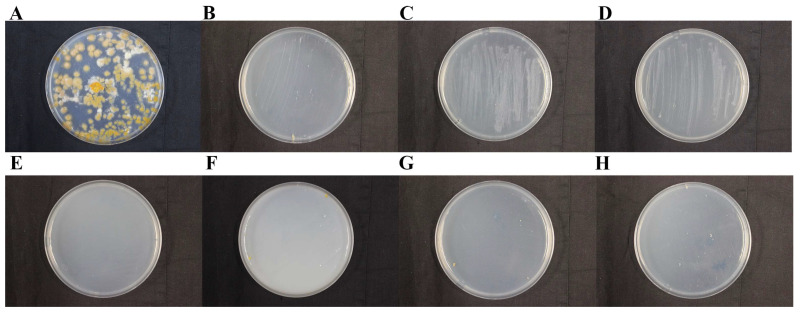
Inhibition effect of various fungicides on *D. stillatus* (DTT1): (**A**) H_2_O; (**B**) 0.375‰ isothiazolinone; (**C**) 0.15‰ isothiazolinone; (**D**) 0.9‰ isothiazolinone; (**E**) 0.5% quaternary ammonium salt; (**F**) 3.5% quaternary ammonium salt; (**G**) 0.35‰ isothiazolinone; and (**H**) 7‰ isothiazolinone. The plates were incubated at 25 °C for 30 days.

**Figure 8:**
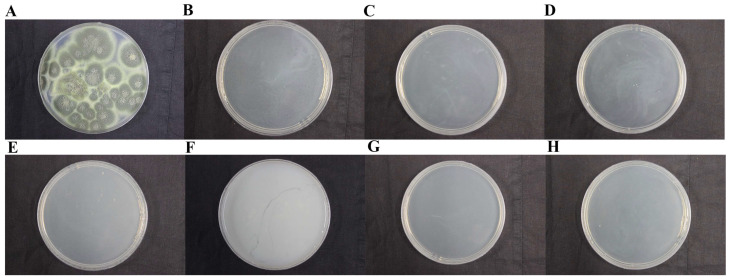
Inhibition effect of various fungicides on T. pinophilus (DTT2): (**A**) H_2_O; (**B**) 0.375‰ isothiazolinone; (**C**) 0.15‰ isothiazolinone; (**D**) 0.9‰ isothiazolinone; (**E**) 0.5% quaternary ammonium salt; (**F**) 3.5% quaternary ammonium salt; (**G**) 0.35‰ isothiazolinone; and (**H**) 7‰ isothiazolinone. The plates were incubated at 28 °C for 10 days.

The fungicidal effects of a fungicide, miconazole nitrate, used in the Dingtao M2 tomb, were tested. The results showed that miconazole nitrate did not inhibit the growth of DTT1 at a concentration of 5%, and a high concentration of miconazole nitrate significantly changed the growth morphogenesis of DTT1. The colony of DTT1 turned gray, with transparent crystals appearing in the colony (Figure S4B). The fungicide K100, currently used by the “Nanhai” No.1 shipwreck, is an isothiazolinone. The results showed that 0.5% of K100 completely inhibited the growth of DTT1 on the PDA medium (Figure S4C). Because of its high price, K100 is not suitable for large-scale onsite use. Therefore, we tested other fungicides (isothiazolinone BC01 and BC14; quaternary ammonium salt BC08) for their inhibitory effects on DTT1 at both conventional and minimum concentrations. These fungicides completely inhibited the growth of DTT1 on the PDA medium (Figure 7). Subsequent experiments showed that these fungicides could also effectively inhibit the growth of DTT2 on PDA (Figure 8).

## 3. Discussion

The Dingtao M2 Tomb is the largest and best-preserved imperial “Huangchangticou” tomb in China, with extremely precious cultural and historical value. Therefore, the preservation work for this site is of utmost importance. For wooden cultural relics, microbial problems are quite common, as seen in the “Nanhai” NO.1 shipwreck [23] and the “Xiaobaijiao” NO.1 shipwreck [24]. However, this was the first time that *Dacrymyces* was discovered on cultural relics. Since the excavation of the M2 tomb, microbial contamination has been present. While various methods have been attempted to eliminate and inhibit microorganisms, such as using blades to remove surface microorganisms, the results have not been optimal. This study investigated and analyzed the microbial diversity on the surface and surrounding environment of the M2 tomb in July 2021 and August 2022. It detected the species composition and population proportion of microorganisms, explored the biodegradation mechanism of dominant fungi, screened suitable fungicides, and provided data to support future conservation investigations and efforts.

In March 2015, a significant number of white plaques appeared on the surface of the Dingtao M2 tomb, and high-throughput sequencing results identified them as *Hypochnicium* [22]. In July 2021, a new type of white plaque emerged on the tomb surface. High-throughput sequencing identified them as *Dacrymyces*. By August 2022, the predominant fungus on the tomb surface had shifted to *Talaromyces*, with *Dacrymyces* making up less than 1% of the composition. Throughout 2015 to 2022, the predominant fungi on the tomb surface changed three times, prompting further exploration into the reasons for these shifts. Community succession among microorganisms is a common phenomenon in natural environments, particularly in decaying wood. Some researchers have observed that soft rot fungi are the pioneers in the decomposition of dead wood, followed by white rot fungi, and eventually brown rot fungi [25,26]. However, the specific succession pattern is heavily influenced by the surrounding environment. It was reported that Basidiomycota often plays a prominent role in the early stages, while the abundance of Ascomycota tends to increase as degradation progresses [27]. The community succession on the tomb surface is in line with this phenomenon, with the predominant fungus changing from the white-rot fungus *Hypochnicium* of Basidiomycota to the brown-rot fungus *Dacrymyces* of Basidiomycota, and then to *Talaromyces* of Ascomycota. Microorganisms present on the surface of the Dingtao M2 Tomb were periodically removed by protective personnel as they reached a certain level of growth. In 2015, the M2 tomb was discovered underground, with its lower level still submerged in water. The tomb’s environment was characterized by high humidity and temperatures, consistently ranging from 10 to 16 °C throughout the year. *Hypochnicium*, being a white-rot fungus with resistance to low temperatures, thrived as the dominant fungus on the tomb’s surface under these conditions. Cultural relic preservation workers used mechanical methods to remove *Hypochnicium* to prevent wood degradation caused by this fungus. Later, the M2 tomb began to be dismantled, and the groundwater was cleared. The tomb’s overall environment underwent significant changes, including reduced humidity, increased temperature, and improved ventilation. There was no obvious colonization of *Hypochnicium* on the tomb’s surface. In 2021, *Dacrymyces* appeared on the surface of the tombs. The reason for this appearance is unclear, but it is speculated that spores may have spread through the air. *Dacrymyces* are brown rot fungi that can colonize the surface of tombs, becoming the dominant fungus. To prevent wood degradation by *Dacrymyces*, the staff removed the affected wood. However, to avoid damaging cultural relics, the removal was not thorough and the tomb’s environment remains unchanged. *Talaromyces* eventually replaced *Dacrymyces* as the predominant fungus. There are three possible reasons for this substitution: first, the wood surface may contain nutrients that *Talaromyces* can use for growth after degradation by *Hypochnicium*, *Dacrymyces*, and other bacteria. The growth rate of *Talaromyces* is faster than that of *Dacrymyces*; therefore, *Talaromyces* occupies an ecological niche. Another possibility is that *Talaromyces* secretes substances that inhibit the growth of *Dacrymyces*. The last possibility is that *Talaromyces* can parasitize *Dacrymyces*, as previous research has reported that *Talaromyces* can parasitize other fungi such as *Aspergillus flavus* and *Monascus* [28]. All three possibilities require further investigation for verification.

Fungi are currently the most effective decomposers of wood, and microbial diseases of the M2 tombs have also been caused by fungi. Dacrymycetaceae species often inhabit dead trees and stumps of flowering plants and gymnosperms. Most species of Dacrymycetaceae are brown rot fungi, capable of degrading significant amounts of lignin. White rot is caused by *Dacrymyces* [29]. It was reported that Dacrymycetaceae is the evolutionary branch with the oldest origin among wood decomposers of Basidiomycota, suggesting that their ancestors were likely the first batch of basidiomycetes capable of degrading wood [30]. The FUNGuild annotation data also showed that *Dacrymyces* were wood-saprophytic fungi, degrading cellulose and lignin, which poses great harm to the M2 tomb. The tomb has been buried underground for over 2000 years, and, after excavation, some elements remain on the surface of the wood, which is different from wood in other natural environments. We took samples of two tomb wood artifacts and identified that these contained significant levels of Fe_3_(PO_4_)_2_·3H_2_O and C_2_CaO_4_·2H_2_O. Using various concentrations of Ca^2+^ and Fe^2+^, we studied the effects of these two ions on the activity of cellulose and lignin-degrading enzymes of *Dacrymyces*. The results showed that the laccase and cellulase activities of *Dacrymyces* increased in environments with high concentrations of Ca^2+^ and Fe^2+^, suggesting that Ca^2+^ and Fe^2+^ contents in tomb wood may be more conducive to wood degradation by *Dacrymyces*.

*Talaromyces* is highly adaptable to various environments and is widely occurring in air, soil, water, and areas inhabited by humans. According to reports, *Talaromyces* has a strong ability to degrade cellulose and is an important fungus used for biomass degradation [31,32]. *T. pinophilus*, *T. purpureogenus*, and *T. verruculosus* can produce highly efficient cellulase to hydrolyze plant cellulose [33,34]. *T. verruculosus* secretes up to nine types of cellulases [35]. Further, *Talaromyces* secretes different enzymes in response to different lignocellulose inducers, including xylanase, β-xylosidase, and arabiofuranosidase [36]. Additionally, some strains, like *T. thermophilus* can produce heat-stable endoxylanase and β-xylosidase [37]. *T. cellulolyticus* and *T. amestolkiae* have a significant number of genes that encode CAZymes [38]. This study also showed that *Talaromyces* DTT2 can utilize cellulose and lignin. Previous reports indicate that most *Talaromyces* species can produce pigments, typically yellow and red. The main components of these pigments are azophilic ketone-like polyketone compounds, and studies showed that these pigments are non-toxic. Some species of *Talaromyces* produce pigments that are only found in their mycelium, such as *T. minioluteus*, *T. ruber*, *T. amestolkiae*, and *T. stollii*, while other species produce pigments that can be secreted outside the cell, such as *T. marneffei*, *T. albobiverticillius*, *T. atroroseus*, *T. rubrifaciens* and *T. purpureogenus* [39,40,41,42]. Therefore, *Talaromyces* may also secrete pigments that could potentially affect cultural relics.

Water is crucial for the growth of fungi [43], and the wood excavated from the M2 tomb contains a large amount of water after being soaked in groundwater. The saturated state of the wood is not only conducive to fungal colonization but also makes subsequent preservation difficult. After excavation, the environment in which the cultural relics are located underwent significant changes. If not properly preserved, the wood undergoes a series of irreparable damages, such as cracking and deformation [44]. Therefore, special attention should be paid to the prevention and control of microbial degradation of the wood. Currently, mechanical methods remain the most direct and effective means for quickly addressing large-scale outbreaks of microorganisms in the M2 tomb. Research has shown that microorganisms growing on the wood surface can cause serious damage to wood; therefore, timely cleaning is necessary. Mechanical methods alone are insufficient to remove plaque, and other physical and chemical methods must be used. Currently, a sterilization pot is equipped on-site to sterilize items contaminated with plaque under high temperatures and pressure, which can effectively prevent the spread of fungi. The rational use of chemical fungicides can effectively inhibit the growth of microorganisms [45]. The in vitro data of the fungicidal investigation confirmed that isothiazolinone fungicides could effectively inhibit the growth of fungal disease. Cultural relics are valuable, and any fungicide used on them should be thoroughly tested in advance. While fungicides can temporarily control microbial problems, further research is needed to determine whether they may cause other issues.

The preservation of cultural relics is a long-term process that requires research on diseased microorganisms. In the future, it is important to conduct extensive research on microbial issues of the M2 tombs to discover more effective preservation methods.

## 4. Materials and Methods

### 4.1. Sample Collection and Microbial Investigation

**Figure 9:**
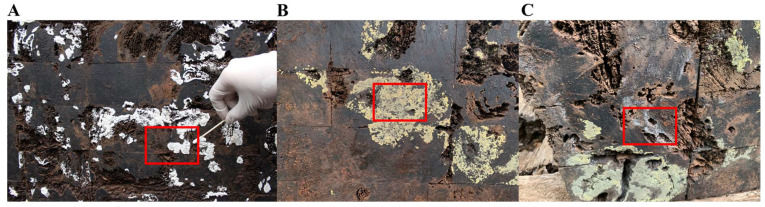
Microbial diseases on Dingtao M2 tomb and its surrounding environment: (**A**) 2021.7 white plaque; (**B**) 2022.8 yellow-green plaque; and (**C**) 2022.8 white mottled plaque.

This study conducted microbial investigations of the M2 tomb during July 2021 and August 2022, finding varying degrees of microbial contamination on the tomb’s surface. In July 2021, during the investigation, the environmental temperature of the tomb was 27.5 °C with a relative humidity of 70%. Many white plaques were discovered on the tomb’s surface (Figure 9A), and green organisms were observed on the earthen area at the tomb’s entrance (Figure S1). Four samples (DTTS1–DTTS4) were collected from the white plaque, one sample (soil) was collected from the earthen area, and two tomb wood samples (DTWood1 and DTWood2) were also collected. In August 2022, during the investigation, the environmental temperature of the tomb chamber was 28 °C with a relative humidity of 76%. This study revealed that the original white plaques on the tomb’s surface disappeared, replaced by more yellow-green plaques (Figure 9B) and a small amount of white spotted plaque (Figure 9C). Three samples (DTM2.1–DTM2.3) were collected from the yellow-green plaque, and one sample (DTM2.4) was collected from the white-spotted plaque. We gently scraped the plaque with a sterile surgical knife and placed it in a sterile EP tube. We then transported the samples to the laboratory for further research.

### 4.2. SEM Observation

Carbon conductive adhesive was used to adhere microbial samples to the surface of the M2 tomb, which were then dried in a drying dish. After drying, the sample was affixed to the SEM sample stage. Gold was sprayed with a 24 mA current for 300 s, the sample was observed using SEM, and images were recorded. The measurement conditions were as follows: EHT: 15.0 kV, WD: 9.6–10.2 mm, and Mag: 0.3KX–5KX.

### 4.3. Metabolome Analysis

The DNeasy PowerSoil Pro Kit (QIAGEN, Hilden, Germany, Cat. No. 47014) was used to extract total DNA from solid samples. The total extracted DNA from samples collected in July 2021 was sent to Majorbio Technology Co., Ltd. (Shanghai, China). The 18S rRNA genes of distinct regions (18S V4) were amplified using specific primers (528F-706R) with barcodes. All PCR mixtures contained 15 µL of the Phusion^®^ High-Fidelity PCR Master Mix (New England Biolabs, lpswich, MA, USA, Cat. No. M0531S), 0.2 µM of each primer and 10 ng of target DNA, and cycling conditions consisted of a first denaturation step at 98 °C for 1 min, followed by 30 cycles at 98 °C (10 s), 50 °C (30 s), and 72 °C (30 s), and a final 5min extension at 72 °C. Sequencing libraries were prepared with the TruSeq^®^ DNA PCR-Free Sample Preparation Kit (Illumina, San Diego, CA, USA, Cat. No.15032317) following the manufacturer’s instructions, and index codes were added. Library quality was assessed using a Qubit@ 2.0 Fluorometer (Thermo Fisher Scientific, Waltham, MA, USA) and an Agilent Bioanalyzer 2100 system. The library was then sequenced on an Illumina NovaSeq platform, generating 250 bp paired-end reads. Paired-end reads were matched to samples based on their unique barcodes and then trimmed to remove the barcodes and primer sequences. The paired-end reads were merged using FLASH (V1.2.7, http://ccb.jhu.edu/software/FLASH (accessed on 10 September 2021)). Quality filtering of raw tags was conducted under specific conditions to obtain high-quality clean tags following the QIIME (V1.9.1, http://qiime.org/scripts/split_libraries_fastq.html (accessed on 10 September 2021)) quality control process. The tags were compared to the Silva database using the UCHIME algorithm (http://www.drive5.com/usearch/manual/uchime_algo.html (accessed on 10 September 2021)) to identify and remove chimeric sequences. The effective tags were then obtained. A sequence analysis was conducted using the Uparse software (Uparse v7.0.1001, http://drive5.com/uparse/ (accessed on 10 September 2021)). Sequences with ≥97% similarity were assigned to the same operational OTUs. A representative sequence from each OTU was selected for further annotation, using the Silva Database (http://www.arb-silva.de (accessed on 10 September 2021)) and the Mothur algorithm to annotate the taxonomic information. The abundance information of OTUs was normalized to a standard sequence number corresponding to the sample with the lowest number of sequences. The raw sequencing data can be accessed at the NCBI Sequence Read Archive (SRA) under the study accession number PRJNA1015011.

### 4.4. Metagenomic Sequencing Analysis

Total genomic DNA was extracted using the DNeasy PowerSoil Pro Kit (QIAGEN, Germany, Cat. No. 47014) and sent to NovoMagic Technology Co., Ltd. (Beijing, China). for sequencing on an Illumina PE150 sequencing platform. The DNA was randomly sheared into short fragments, which were then end-repaired, A-tailed, and ligated using an Illumina adapter. Fragments containing adapters were PCR-amplified, size selected, and purified. The library was checked using Qubit and real-time PCR for quantification, and a bioanalyzer was used for size distribution detection. Quantified libraries were pooled and sequenced on Illumina platforms according to the effective library concentration and the amount of data required. Readfq (V8, https://github.com/cjfields/readfq (accessed on 9 August 2022)) was used to preprocess the raw data from the Illumina sequencing platform to obtain clean data for subsequent analysis. The raw sequencing data are accessible at the NCBI Sequence Read Archive (SRA) under the study accession number PRJNA1015011.

### 4.5. X-ray Fluorescence

The relative contents of various elements in the wood were determined using X-ray fluorescence (XRF). The wood blocks were crushed with a grinder and then placed in a ventilated area to dry before being filtered through an 80-mesh sieve. The filtered wood powder was then placed in the testing circle of the instrument and pressed into a compact plane. The content of each element in the wood powder was determined using XRF. The measurement conditions were as follows: tube voltage of 50 KV, output current of 4 mA, and rated power of 200 W.

### 4.6. X-ray Diffraction

The compounds present in the wood samples were determined using a diffractometer (XRD). The wood blocks were crushed with a grinder, then placed in a ventilated area for drying, and filtered through an 80-mesh sieve. The filtered wood powder was then placed in a sample tank and pressed onto a flat surface for testing. The measurement conditions were as follows: Cu target, power of 9 kW, scanning speed of 20°/min, and 2θ scanning range of 5–80°.

### 4.7. Detection of Fungal Cellulose Lignin Degradation Capability

For cellulose detection, the fungi were grown in the CMC medium (0.2% NaNO_3_, 0.05% KCl, 0.1% K_2_HPO_4_, 0.05% MgSO_4_, 0.2% CMC, 0.2% peptone, 2% agar) at 28 °C for 7 days. Then, 5 mL of iodine-potassium iodide solution was added to the plate and incubated for 5 min at room temperature in the dark. Fungi that degrade cellulose will produce transparent rings. For lignin detection, the fungi were grown in a PDA guaiacol medium (1.2% potato extract, 2% glucose, 0.04% guaiacol, 2% agar) at 28 °C for 7 days. If fungi can utilize lignin, they produce a brown reaction.

### 4.8. Detection of Cellulose and Lignin Degrading Enzymes

The fungi were grown in 100 mL of a PD medium (1.2% potato extract, 2% glucose) at 25 °C for 5 days. Next, 500 µL of this fungal suspension was inoculated into fresh 50 mL PD medium and incubated for 3–15 days, conducting three parallel experiments in each group. On various days, the culture was centrifuged, and the supernatant was collected to detect protein content and enzyme activity. The protein content was determined using the BCA protein assay kit (Solarbio, Beijing, China, Cat. No. PC0020), which works by proteins reducing Cu^2+^ to Cu^+^ under alkaline conditions, reacting with BCA reagents to form a purple-blue complex with a maximum absorption at 562 nm. To assay manganese peroxidase (MnP) activity, a manganese peroxidase assay kit (Solarbio, Beijing, China, Cat. No. BC1620) was used, which oxidizes guaiacol to tetra methoxyphenol with an absorption peak at 465 nm. A lignin peroxidase assay kit (Solarbio, Beijing, China, Cat. No. BC1610) was used to assess the lignin peroxidase (LiP) activity, which oxidizes resveratrol to veratraldehyde and was measured at 310 nm. The laccase (Lac) activity was determined using a laccase assay kit (Grace Biotechnology, Suzhou, China, Cat. No. G0541W), where Lac decomposes the substrate 2,2′-Azinobis-(3-ethylbenzthiazoline-6-sulphonate) (ABTS) to produce ABTS free radicals with absorbance at 420 nm. Lastly, the cellulase activity was assayed using the cellulase assay kit (Bioss, Beijing, China, Cat. No. AK196), where cellulase catalyzes the degradation of cellulose to produce reducing sugars with a maximum absorption peak at 550 nm using carboxymethyl cellulose as a reaction substrate. A statistical analysis was conducted using a Standard Deviation (SD) test.

### 4.9. Optimization of the Growth Conditions

CaCl_2_ and FeSO_4_ were used to optimize Ca^2+^ and Fe^2+^ in the assay conditions for the activity of cellulose and lignin-degrading enzymes. The PD liquid culture medium was utilized, with Fe^2+^ concentrations set at 0, 25, 50, and 100 mg/L and Ca^2+^ concentrations set at 0, 50, 100, and 200 mg/L. Fungi were inoculated into 100 mL of a PD medium and incubated at 25 °C for 5 days to produce the fungal suspension. A total of 500 µL of the fungal suspension was then inoculated into fresh PD mediums, with three parallel experiments conducted in each group. The fungal suspensions grown under various culture conditions were centrifuged, and the supernatant was collected to measure protein content and enzyme activity. Statistical analysis was conducted using an SD test.

### 4.10. Fungicidal Experiment

To screen effective fungicides, fungicidal experiments were used (Table 2). First, the fungicide was diluted separately with water, and then 1 mL of the solution was added to 19 mL of a melted PDA culture medium. The mixture was poured into a culture dish and allowed to solidify. Fungi were inoculated into 100 mL of a PD medium and incubated at 25 °C for 5 days to obtain fungal suspension. A total of 200 µL of this fungal suspension was inoculated into a PD medium containing fungicide. A sterile cotton swab was used to evenly coat the fungi on the medium and incubated as described above, with three biological replicates.

## 5. Conclusions

This study revealed significant fungal colonization on the surface of the M2 tomb. In July 2021, *Dacrymyces* was the predominant fungus, while *Talaromyces* took over by August 2022. Both fungi have strong cellulose and lignin degradation activities. The tomb wood contained high amounts of Fe^2+^ and Ca^2+^, leading to higher activities of cellulose and lignin-degrading enzymes by *Dacrymyces*. The isothiazolinone fungicide showed a strong inhibitory effect on the two dominant fungi.

## Figures and Tables

**Table 1:** The parameters of cellulose and lignin degradation enzymes of DTT1.

Enzyme	Maximum Enzyme Activity (U/mL)	Maximum Specific Enzyme Activity (U/mg)	Cultivation Time (Days)
MnP	0.4958	0.2057	10
Lip	2.3225	0.9760	8
Lac	0.1778	0.0725	12
Cellulase	104.6331	42.6830	13

**Table 2:** Fungicides used in this study.

Fungicide	Main Fungicidal Components	Concentration (*m*/*v*)
K100	0.75% Isothiazolinone	0.5%
Miconazole Nitrate	Miconazole Nitrate	5%
BC01	3% Isothiazolinone	0.05%, 0.3%
BC08	Quaternary ammonium salt, 1.02 g/mL	0.5%, 3.5%
BC14	14% Isothiazolinone	0.025%, 0.5%

## Data Availability

The raw sequencing data can be accessed at the NCBI Sequence Read Archive (SRA) under the study accession number PRJNA1015011.

## Supplementary Materials

The following supporting information can be downloaded at https://www.mdpi.com/article/10.3390/ijms252212270/s1.

## References

[1] Wu Q., Zhang B., Hu Y. (2023). Comparison and research progress of protein detection technology for cultural relic materials. Coatings.

[2] Zhou Q., Yan W. (2011). Oscillation response of museum cultural relics under earthquake by shaking table tests. Appl. Mech. Mater..

[3] Pruvost M., Bellone R., Benecke N., Sandoval-Castellanos E., Cieslak M., Kuznetsova T., Morales-Muñiz A., O’Connor T., Reissmann M., Hofreiter M. (2011). Genotypes of predomestic horses match phenotypes painted in Paleolithic works of cave art. Proc. Natl. Acad. Sci. USA.

[4] Sterflinger K., Pinzari F. (2012). The revenge of time: Fungal deterioration of cultural heritage with particular reference to books, paper and parchment. Environ. Microbiol..

[5] Sterflinger K., Piñar G. (2013). Microbial deterioration of cultural heritage and works of art—Tilting at windmills?. Appl. Microbiol. Biotechnol..

[6] Piñar G., Sterflinger K., Pinzari F. (2015). Unmasking the measles-like parchment discoloration: Molecular and microanalytical approach. Environ. Microbiol..

[7] Kakakhel M.A., Wu F., Gu J., Feng H., Shah K., Wang W. (2019). Controlling biodeterioration of cultural heritage objects with biocides: A review. Int. Biodeterior. Biodegrad..

[8] Farooq M., Hassan M., Gull F. (2015). Mycobial deterioration of stone monuments of Dharmarajika, Taxila. J. Microbiol. Exp..

[9] Meng H., Katayama Y., Gu J. (2017). More wide occurrence and dominance of ammonia-oxidizing oarchaea than bacteria at three Angkor sandstone temples of Bayon, Phnom Krom and Wat Athvea in Cambodia. Int. Biodeterior. Biodegrad..

[10] Elam J., Björdal C.G. (2022). Degradation of wood buried in soils exposed to artificially lowered groundwater levels in a laboratory setting. Int. Biodeterior. Biodegrad..

[11] Pearsall D.M. (2015). Paleoethnobotany: A Handbook of Procedures.

[12] Beccaccioli M., Moricca C., Faino L., Reale R., Mineo M., Reverberi M. (2023). The Neolithic site La Marmotta: DNA metabarcoding to identify the microbial deterioration of waterlogged archeological wood. Front. Microbiol..

[13] Liu Z., Fu T., Hu C., Shen D., Macchioni N., Sozzi L., Chen Y., Liu J., Tian X., Ge Q. (2018). Microbial community analysis and biodeterioration of waterlogged archaeological wood from the Nanhai No. 1 shipwreck during storage. Sci. Rep..

[14] Björdal C.G., Nilsson T., Daniel G. (1999). Microbial decay of waterlogged archaeological wood found in Sweden Applicable to archaeology and conservation. Int. Biodeterior. Biodegrad..

[15] Capretti C., Macchioni N., Pizzo B., Galotta G., Giachi G., Giampaola D. (2008). The characterization of waterlogged archaeological wood: The three roman ships found in Naples (Italy). Archaeometry.

[16] Schwarze F. (2007). Wood decay under the microscope. Fungal Biol. Rev..

[17] Björdal C.G. (2012). Microbial degradation of waterlogged archaeological wood. J. Cult. Herit..

[18] Rytioja J., Hildén K., Yuzon J., Hatakka A., Vries R.P., Mäkelä M.R. (2014). Plant-polysaccharide-degrading enzymes from Basidiomycetes. Microbiol. Mol. Biol. Rev..

[19] Medie F.M., Davies G.J., Drancourt M., Henrissat B. (2012). Genome analyses highlight the different biological roles of cellulases. Nat. Rev. Microbiol..

[20] Obeng E.M., Adam S.N.N., Budiman C., Ongkudon C.M., Maas R., Jose J. (2017). Lignocellulases: A review of emerging and developing enzymes, systems, and practices. Bioresour. Bioprocess..

[21] Xu S., Ma Q., Xu S. (2022). Fuzzy comprehensive evaluation of the compatibility of restoration materials—Case study in the rammed earth restoration of the M2 Han tomb in Dingtao, Shandong Province. J. Cult. Herit..

[22] Liu Z., Wang Y., Pan X., Ge Q., Ma Q., Li Q., Fu T., Hu C., Zhu X., Pan J. (2017). Identification of fungal communities associated with the biodeterioration of waterlogged archeological wood in a Han dynasty tomb in China. Front. Microbiol..

[23] Han Y., Huang X., Wang Y., Du J., Ma K., Chen Y., Li N., Zhang Z., Pan J. (2021). Fungal community and biodeterioration analysis of hull wood and its storage environment of the Nanhai No. 1 shipwreck. Front. Microbiol..

[24] He Y., Li W., Lu X., Xu C., Jin T., Lin G. (2021). Chinese export porcelain in the middle Qing Dynasty: Study on the blue-and-white porcelains excavated from the Xiaobaijiao I shipwreck. J. Archaeol. Sci. Rep..

[25] Rajala T., Peltoniemi M., Hantula J., Mäkipää R., Pennanen T. (2011). RNA reveals a succession of active fungi during decay of Norway spruce logs. Fungal Ecol..

[26] Bani A., Pioli S., Ventura M., Panzacchi P., Borruso L., Tognetti R., Tonon G., Brusetti L. (2018). The role of microbial community in the decomposition of leaf litter and deadwood. Appl. Soil Ecol..

[27] Gómez-Brandón M., Probst M., Siles J.A., Peintner U., Bardelli T., Egli M., Insam H., Ascher-Jenull J. (2020). Fungal communities and their association with nitrogen-fixing bacteria affect early decomposition of Norway spruce deadwood. Sci. Rep..

[28] Sun J., Ruan Y., Jin S., Wang L. (2021). The importance of *Talaromyces* and its taxonomic studies. J. Fungal Res..

[29] Nagy L.G., Riley R., Tritt A., Adam C., Daum C., Floudas D., Sun H., Yadav J.S., Pangilinan J., Larsson K.H. (2015). Comparative genomics of early-diverging mushroom-forming fungi provides insights into the origins of lignocellulose decay capabilities. Mol. Biol. Evol..

[30] Floudas D., Binder M., Riley R., Barry K., Blanchette R.A., Henrissat B., Martínez A.T., Otillar R., Spatafora J.W., Yadav J.S. (2012). The Paleozoic origin of enzymatic lignin decomposition reconstructed from 31 fungal genomes. Science.

[31] Fujii T., Hoshino T., Inoue H., Yano S. (2013). Taxonomic revision of the cellulose degrading fungus *Acremonium cellulolyticus* nomen nudum to *Talaromyces* based on phylogenetic analysis. FEMS Microbiol. Lett..

[32] Houbraken J., Spierenburg H., Frisvad J.C. (2012). Rasamsonia, a new genus comprising thermotolerant and thermophilic *Talaromyces* and *Geosmithia* species. Antonie Van Leeuwenhoek.

[33] Pol D., Laxman S., Rao M. (2012). Purification and biochemical characterization of endoglucanase from *Penicillium pinophilum* MS 20. Indian J. Biochem. Biophys..

[34] Maeda R.N., Barcelos C.A., Anna L.M.M., Pereira N. (2013). Cellulase production by *Penicillium funiculosum* and its application in the hydrolysis of sugar cane bagasse for second generation ethanol production by fed batch operation. J. Biotechnol..

[35] Morozova V.V., Gusakov A., Andrianov R.M., Pravilnikov A.G. (2010). Cellulases of *Penicillium verruculosum*. Biotechnol. J..

[36] Goyari S., Devi S.H., Bengyella L., Khan M., Sharma C.K., Kalita M.C., Talukdar N.C. (2015). Unveiling the optimal parameters for cellulolytic characteristics of *Talaromyces verruculosus* SGMNPf3 and its secretory enzymes. J. Appl. Microbiol..

[37] Romdhane I.B.B., Achouri I.M., Belghith H. (2010). Improvement of highly thermostable xylanases production by *Talaromyces thermophilus* for the agro-industrials residue hydrolysis. Appl. Biochem. Biotechnol..

[38] Liao H., Zheng H., Li S., Wei Z., Mei X., Ma H., Shen Q., Xu Y. (2015). Functional diversity and properties of multiple xylanases from *Penicillium oxalicum* GZ-2. Sci. Rep..

[39] Yilmaz N., Houbraken J., Hoekstra E.S., Frisvad J.C., Visagie C.M., Samson R.A. (2012). Delimitation and characterisation of *Talaromyces purpurogenus* and related species. Persoonia.

[40] Frisvad J.C., Yilmaz N., Thrane U., Rasmussen K.B., Houbraken J., Samson R.A. (2013). *Talaromyces atroroseus*, a new species efficiently producing industrially relevant red pigments. PLoS ONE.

[41] Bhatnagar S., Kobori T., Ganesh D., Aoyagi H. (2022). Fungal pigment–assisted silver nanoparticle synthesis and their antimicrobial and cytotoxic potential. Plant Second. Metab. Eng..

[42] Lebeau J., Petit T., Fouillaud M., Dufossé L., Caro Y. (2020). Alternative extraction and characterization of nitrogen-containing azaphilone red pigments and ergosterol derivatives from the marine-derived fungal *Talaromyces* sp. 30570 strain with industrial relevance. Microorganisms.

[43] Blanchette R.A. (2000). A review of microbial deterioration found in archaeological wood from different environments. Int. Biodeterior. Biodegrad..

[44] Björdal C.G., Rönnby J. (2023). Evaluation of in situ preservation method applied at a terrestrial archaeological shipwreck site by use of sacrificial wood samples installed for 25 years. Int. Biodeterior. Biodegrad..

[45] Antonelli F., Bartolini M., Plissonnier M.L., Esposito A., Galotta G., Ricci S., Davidde Petriaggi B., Pedone C., Di Giovanni A., Piazza S. (2020). Essential oils as alternative biocides for the preservation of waterlogged archaeological wood. Microorganisms.

