# Supplementary material for: Microbial Diversity and Biodegradation Mechanism of Microorganisms in the Dingtao M2 Tomb
Source: Int J Mol Sci. 2024 Nov 15;25(22):12270. doi: 10.3390/ijms252212270 (PMC11595116; doi:10.3390/ijms252212270)
Supplement: Supplementary file 1 [file ijms-25-12270-s001.zip › Supplementary materials.pdf]

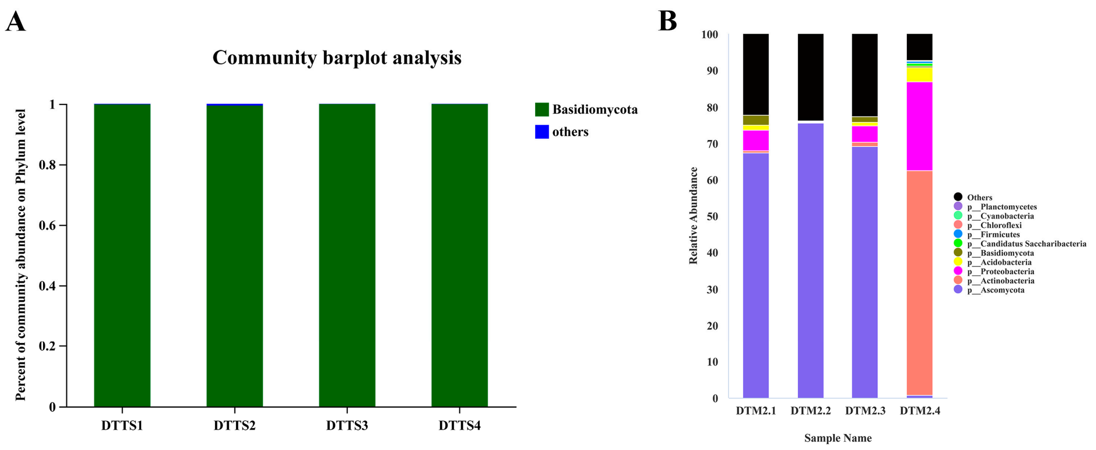

Figure S1. Composition of microbial population on the surface of the Dingtao M2 tomb

(A) 2021.7 Relative abundance of eukaryote at the phylum level; (B) 2022.8 Relative abundance of microorganisms at the phylum level.

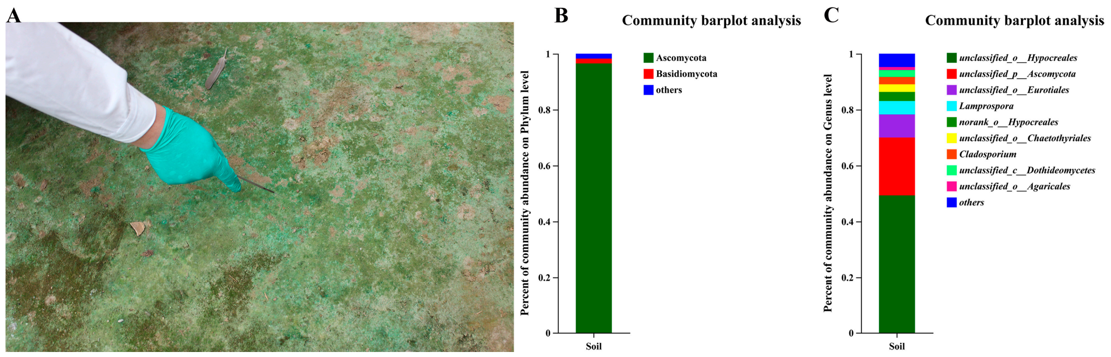

Figure S2. Composition of eukaryotic population of the surroundings (July 2021)

(A) Surrounding earthen ruins of the tomb; (B) Relative abundance of eukaryote of soil sample at the phylum level; (C) Relative abundance of eukaryote of soil sample at the genus level.

Table S1. Annotation results of wood degradation related functions in KEGG database

K0	relative abundance (%)	description
K05349	0.0679	bglX | beta-glucosidase
K01187	0.0476	malZ | alpha-glucosidase
K01179	0.0205	E3.2.1.4 | endoglucanase
K01181	0.0110	E3.2.1.8, xynA | endo-1,4-beta-xylanase
K05350	0.0077	bglB | beta-glucosidase
K19668	0.0072	CBH2, cbhA | cellulose 1,4-beta-cellobiosidase
K01180	0.0071	E3.2.1.6 | endo-1,3(4)-beta-glucanase
K01188	0.0060	E3.2.1.21 | beta-glucosidase
K01225	0.0028	CBH1 | cellulose 1,4-beta-cellobiosidase
K20542	0.0008	bcsZ | endoglucanase

Table S2. Annotation results of wood degradation related functions in eggNOG database

OG_Description	relative abundance (%)
beta-glucosidase	0.0161
mannosyloligosaccharide glucosidase	0.0159
Multi-copper polyphenol oxidoreductase laccase	0.0040
endoglucanase	0.0032
alphaglucosidase	0.0006
Cellulase (glycosyl hydrolase family 5)	0.0003
endoglucanase family 5 glycoside hydrolase	0.0003

Table S3. X-ray fluorescence detection data of tomb wood

element	DTWood.1 (%)	DTWood.2 (%)
Fe	65.773	65.194
Ca	20.176	24.302
Si	3.968	4.739
S	2.483	2.672
Mn	2.012	0.850
Sr	1.508	1.372
K	0.649	0.296
Ti	0.402	0.575

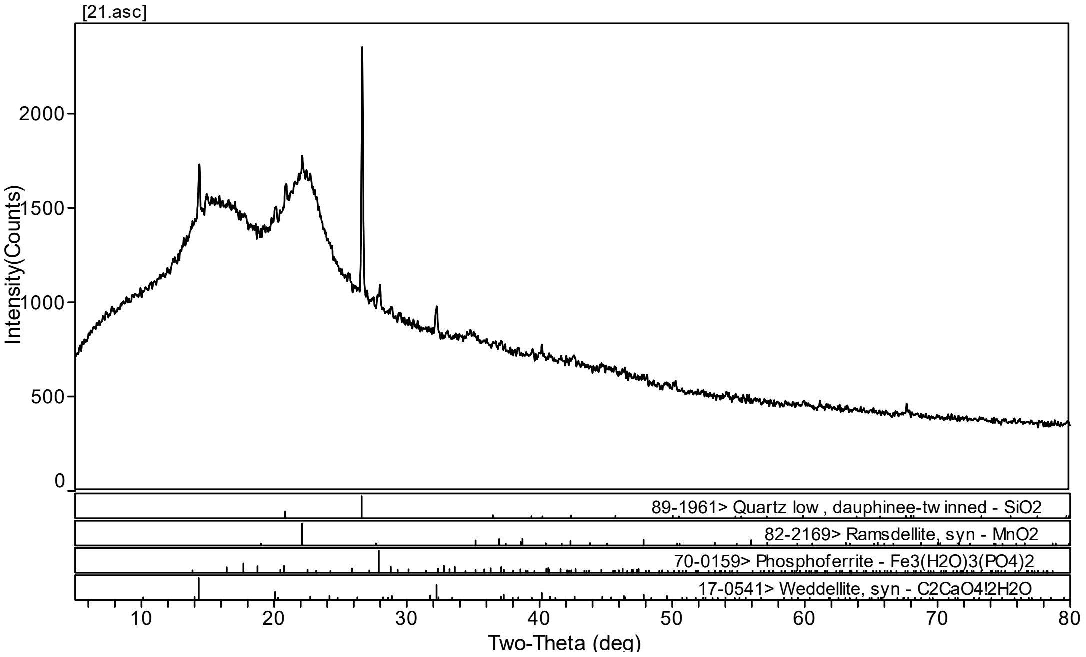

Figure S3. X-ray diffraction results of tomb wood

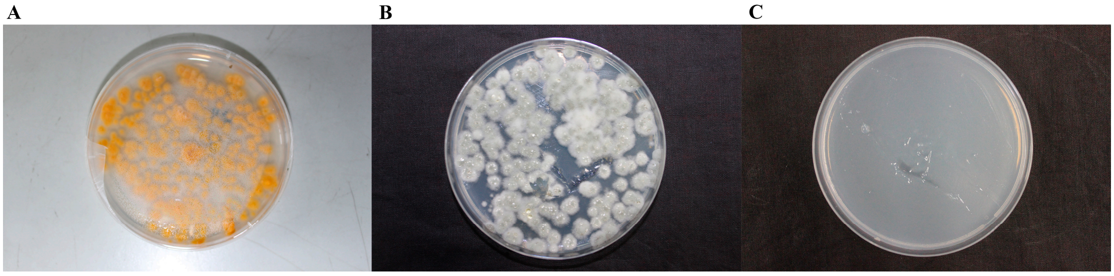

Figure S4. Inhibition effect of different microbicide on *D. stillatus* DTT1
Incubate at 25 ℃ for 10 days. (A) Negative control; (B) 5% Miconazole nitrate; (C) 0.5% K100.
